# Supplementary material for: Droplet Microfluidics Enables Tracing of Target Cells at the Single-Cell Transcriptome Resolution
Source: Bioengineering (Basel). 2022 Nov 10;9(11):674. doi: 10.3390/bioengineering9110674 (PMC9687293; doi:10.3390/bioengineering9110674)
Supplement: Supplementary file 1 [file bioengineering-09-00674-s001.zip › bioengineering-1959441-supplementary.pdf]

bioengineering

MDPI

Article

Droplet Microfluidics Enables Tracing of Target Cells at the Single-Cell Transcriptome Resolution

Yang Liu [1,†], Shiyu Wang [1,2,†], Menghua Lyu [1,2,†], Run Xie [3], Weijin Guo [4], Ying He [5], Xuyang Shi [1,2], Yang Wang [1], Jingyu Qi [1], Qianqian Zhu [1], Hui Zhang [1], Tao Luo [6], Huaying Chen [7], Yonggang Zhu [7], Xuan Dong [1], Zida Li [3], Ying Gu [1], Longqi Liu [1,8,*], Xun Xu [1,9,*] and Ya Liu [1,10,*]

1 BGI-Shenzhen, Shenzhen 518083, China
2 College of Life Sciences, University of Chinese Academy of Sciences, Beijing 100049, China
3 Department of Biomedical Engineering, School of Medicine, Shenzhen University, Shenzhen 518060, China
4 Department of Biomedical Engineering, Shantou University, Shantou 515063, China
5 Department of Gynaecological Oncology, Cancer Hospital Chinese Academy of Medical Sciences, Shenzhen Center, Shenzhen 518116, China
6 Department of Mechanical & Electrical Engineering, Xiamen University, Xiamen 361101, China
7 School of Mechanical Engineering and Automation, Harbin Institute of Technology, Shenzhen, Shenzhen 518055, China
8 Shenzhen Bay Laboratory, Shenzhen 518000, China
9 Guangdong Provincial Key Laboratory of Genome Read and Write, BGI-Shenzhen, Shenzhen 518120, China
10 Shenzhen Key Laboratory of Single-Cell Omics, BGI-Shenzhen, Shenzhen 518100, China
* Correspondence: liulongqi@genomics.cn (L.L.); xuxun@genomics.cn (X.X.); liuya1@genomics.cn (Y.L.)
† These authors contributed equally to this work.

Additional Experimental Section

Details of Chip Design and Fabrication

In brief, the PDMS base and curing agent were mixed at the weight ratio of 10:1, poured onto the mold, and incubated at 80 °C for 1 hour to facilitate the solidification. PDMS chips were peeled off from the model and cut along the chips' outline. In Chip A, one oil inlet hole and two cell/beads inlet holes were made with hole punchers with 5 and 10 mm diameters. In Chip B and Chip C, all inlet and outlet positions were punched into 1 mm diameter holes. Then, these microfluidic chips were fabricated by commonly used materials of PDMS and glass and noted that the surface of PDMS is more hydrophobic than glass. Since Chip A is used to generate water-in-oil droplets, the substrate requires sufficient hydrophobicity to stabilize droplets and prevent uncontrolled coalescence. Chip A was heated for at least 24 h at 60 °C to meet hydrophobic performance. Therefore, the PDMS layer would be a more appropriate selection for the substrate of Chip A. As for Chip B and Chip C, the electrodes were fabricated by infusing melted solder wires into the pre-designed channels on a 90 °C hot plate and then conducted further curation at room temperature. For Chip C, the electrodes were laid at the opposite side of the injection across channels for droplet flowing.

Microscope Imaging

Bright-field images were captured by the Olympus microscope (IX71, Japan) with a high-speed camera (DP26, Olympus, Japan), the pictures of fluorescence-labeled TCR-T cells were acquired using the inverted fluorescence microscope (IX73, Olympus, Japan). Droplets were dropped on a glass slide and imaged with a 10× objective lens of an inverted microscope. Then, these pictures were automatically quantified by ImageJ software to acquire statistical data.

Data Analyzing

The FASTQ data generated by a sequencer were processed by the PISA pipeline (https://github.com/single-cell-BGI/scRNA-pipe), and a standard format of gene expression matrix was generated with reference to GRCh38 human genome for downstream analysis. The gene expression matrix was processed as input for Seurat software (https://github.com/satijalab/seurat). Genes expressed in <3 cells and cells with <200 genes were filtered out. To minimize the batch effect among samples, all samples' analyses were treated by the reciprocal PCA pipeline (parameter: dims = 20) in Seurat to merge into an integrated dataset. The first 30 PCs were summarized to construct an SNN network, and the distance between cells and clusters was calculated by the Louvain algorithm. Then, dimensional reduction and illustration of cells in 2-dimension space were performed with UMAP.

Differential Expressed Genes (DEGs), Gene Ontology (GO) Enrichment, and Cell Cycle Analyses

Identification of DEGs among clusters by "FindAllMarkers" function in Seurat revealed the heterogeneity among TCR-T cells. Genes with adjusted $p < 0.01$ and Foldchange > 2 were defined as DEGs. Then, signaling pathways involved by these DEGs were estimated by GO analyses relying on the function "compare Cluster" (fun ="enrichGO,'' pvalue Cut off = 0.1, pAdjust Method = "BH'', OrgDb = "org.Hs.eg.db,ontBP") of ChIPseeker R package (v.1.22.1), and visualized by R package "clusterProfiler" [1]. The cell cycle analysis and regression were performed with the function "CellCycleScoring" and "ScaleData" in Seurat.

Reference

1. Wu, T.; Hu, E.; Xu, S.; Chen, M.; Guo, P.; Dai, Z.; Feng, T.; Zhou, L.; Tang, W.; Zhan, L. clusterProfiler 4.0: A universal enrichment tool for interpreting omics data. *Innovation* **2021**, *2*, 100141.

Table S1. Materials.

Reagent	Supplier	Catalog Number
Celltracker, green CMFDA dye	Invitrogen	C7025
Dulbecco's Modified Eagle Medium (DMEM)	Gibco	12430054
Dulbecco's phosphate-buffered saline (DPBS)	Gibco	C14190500BT
Droplet Generation Oil for EvaGreen	BIO-RAD	1864005
FEP Tubing, 1/16 inch × 0.25 mm, 10 m	DOLOMITE	3200063
Fetal bovine serum (FBS)	Gibco	192-1005PJ
F68	Pluronic	24040-032
Ficoll-PM400	Cytival	17030010
Micro tubing (inner dimensions: 0.38mm, outer dimension: 1.09mm)	Scientific Commodities	BB31695-PE/2
Negative photoresist SU-8	Microchem	SU-8 3050
Penicillin-Streptomycin (P/S)	Gibco	15140-122

Polydimethylsiloxane (PDMS)	Dow Corning	02085925
Qubit dsDNA HS assay kit	Invitrogen	Q32851
RPMI1640 medium	Gibco	C22400500BT
Silicon (4 inch)	Zhejiang JRH Technology Co., Ltd.	
Single-cell RNA sequencing kit	BGI	
Tris(3-hydroxypropyl)phosphine (THPP)	Sigma-Aldrich	777854
1H,1H,2H,2H–perfluoro-1-octanol (PFO)	Sigma-Aldrich	370533
2 mL collection tube	Corning	430659
1 ml syringe	AndeMed	
10 ml syringe	AndeMed	
30 ml syringe	BD	302833

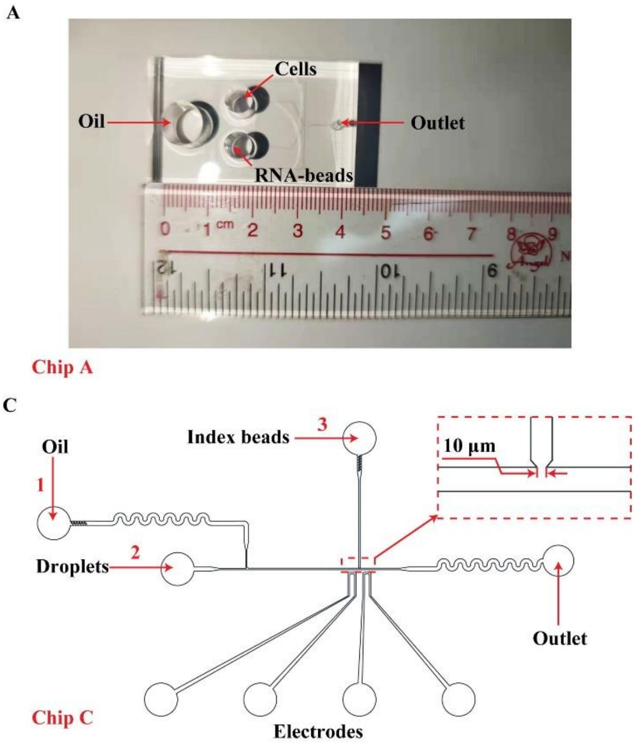

Figure S1. The detailed design of microfluidic chips. (A) Design schematic diagram of droplet generation chip (Chip A) and the amplified view of local details. (B) Real-time recording of droplet generation by negative pressure. Scale bar: 100 μm. (C,D) Design schematic diagram and photograph of droplet injection chip. Scale bar: 100 μm.

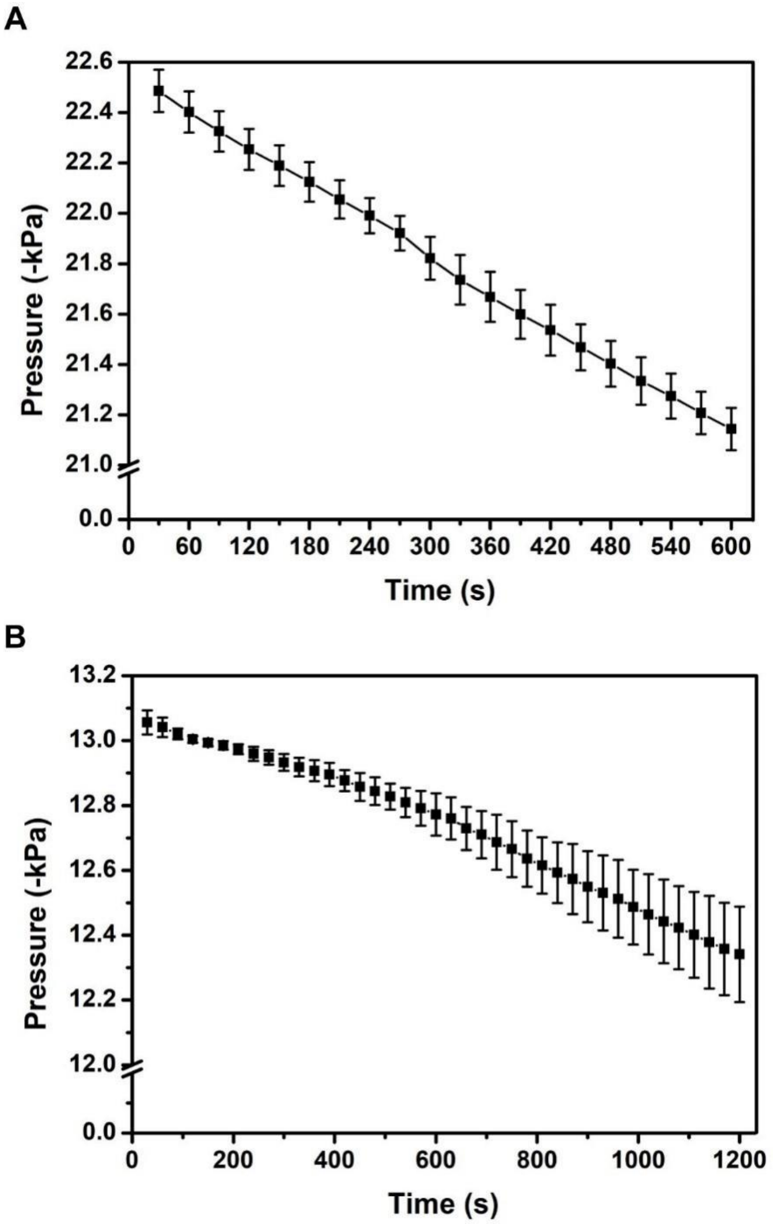

Figure S2. Stability test of negative pressure on microfluidic chips ((A): Chip A; (B): Chip C) N = 3.

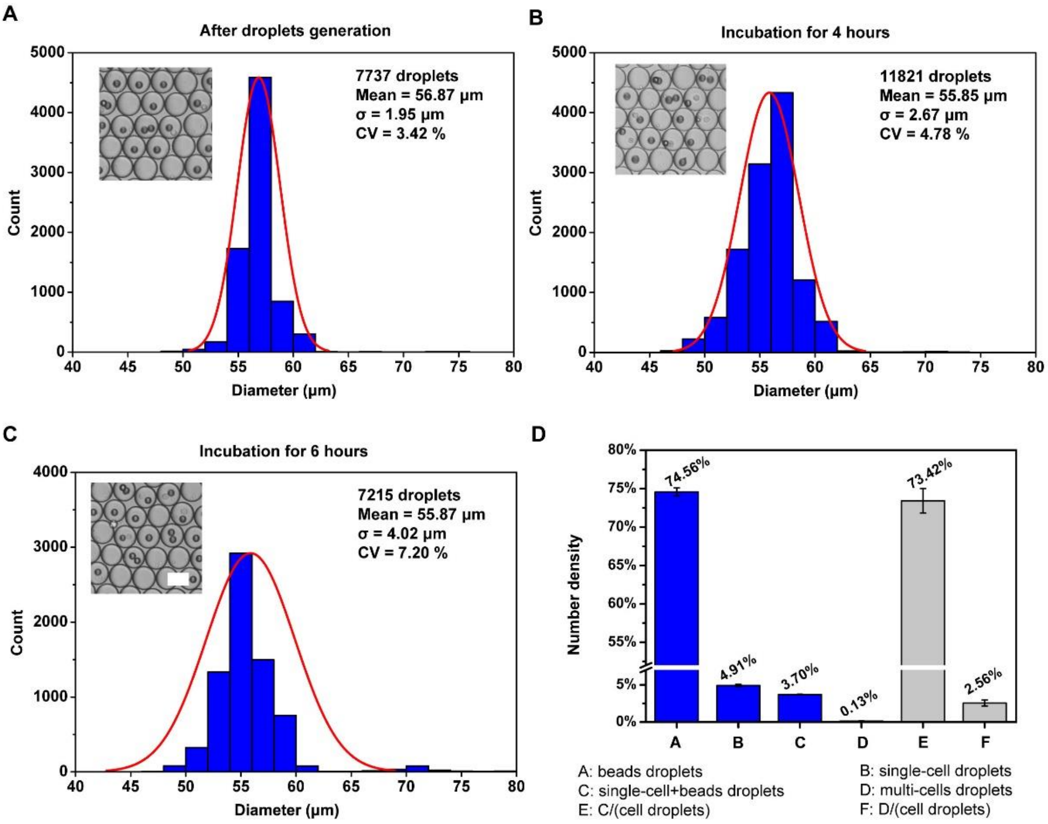

Figure S3. Statistics of the droplet diameter and encapsulation rate of RNA beads. (A) The diameter distribution of 7737 droplets after droplet generation. (B) The diameter distribution of droplets after 4 hours of incubation. (C) The diameter distribution of 7215 droplets after 6 hours of incubation. Scale bar: 50 μm. (D) The proportional distribution of different types of droplets. N = 17113 droplets from three independent experiments.

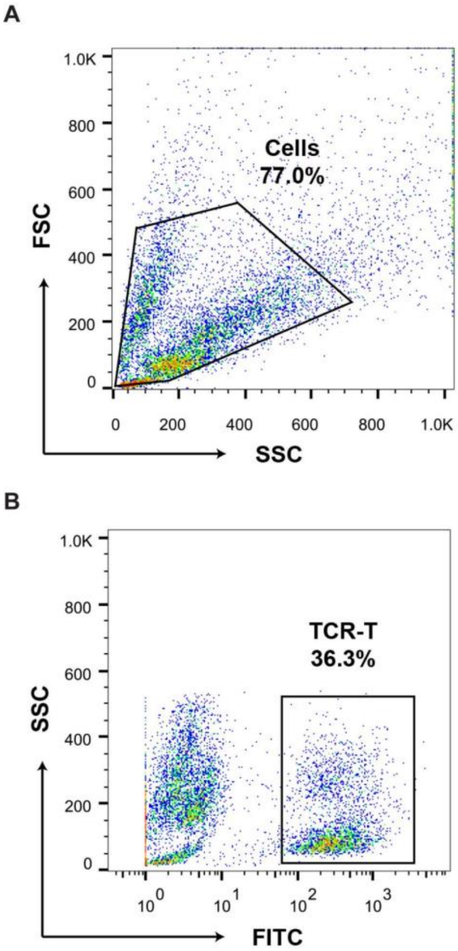

Figure S4. Flow cytometry for the identification of TCR-T cells. (A) SSC-FSC plot of cells. (B) Gating strategy of TCR-T cells based on the CellTracker CMFDA fluorescence. SSC: Side scatter; FSC: Forward scatter.

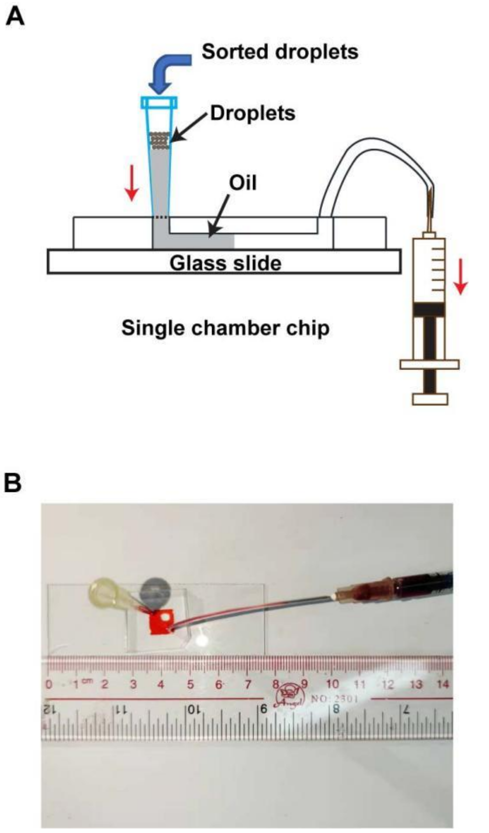

Figure S5. Sorted droplet collector. (A) Schematic diagram of the droplet collector. The sorted droplets in oil flow into the "blue" container that connects a 1mL syringe. As the droplets float on top, the oil should be removed from the bottom by pulling the syringe to avoid droplets flowing out. (B) A picture of the droplet collector. The red buffer was used to indicate components and their connection manner.

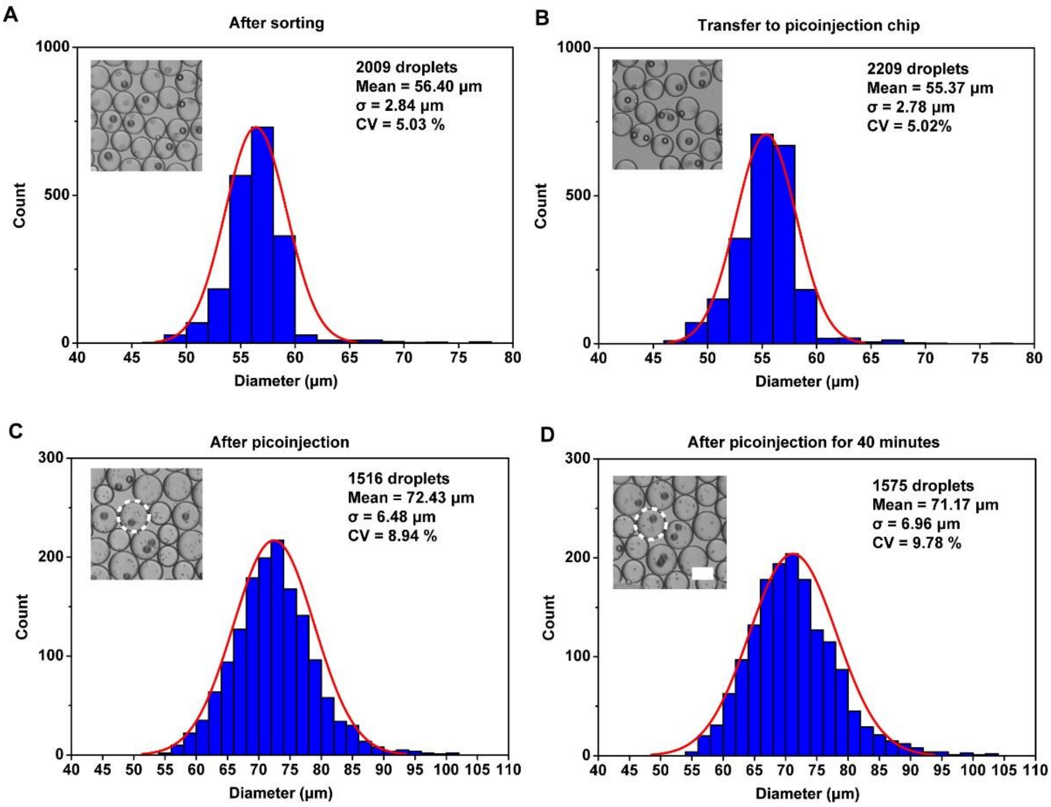

Figure S6. Statistics of the droplet diameter in different conditions. (A) The diameter distribution of 2009 droplets after sorting. (B) The diameter distribution of 2209 droplets after transferring to picoinjection chip. (C) The diameter distribution of droplets after picoinjection. The droplet injected by lysis buffer is circled. (D) The diameter distribution of 1575 droplets after picoinjection for 40 minutes. Scale bar: 50 μm.

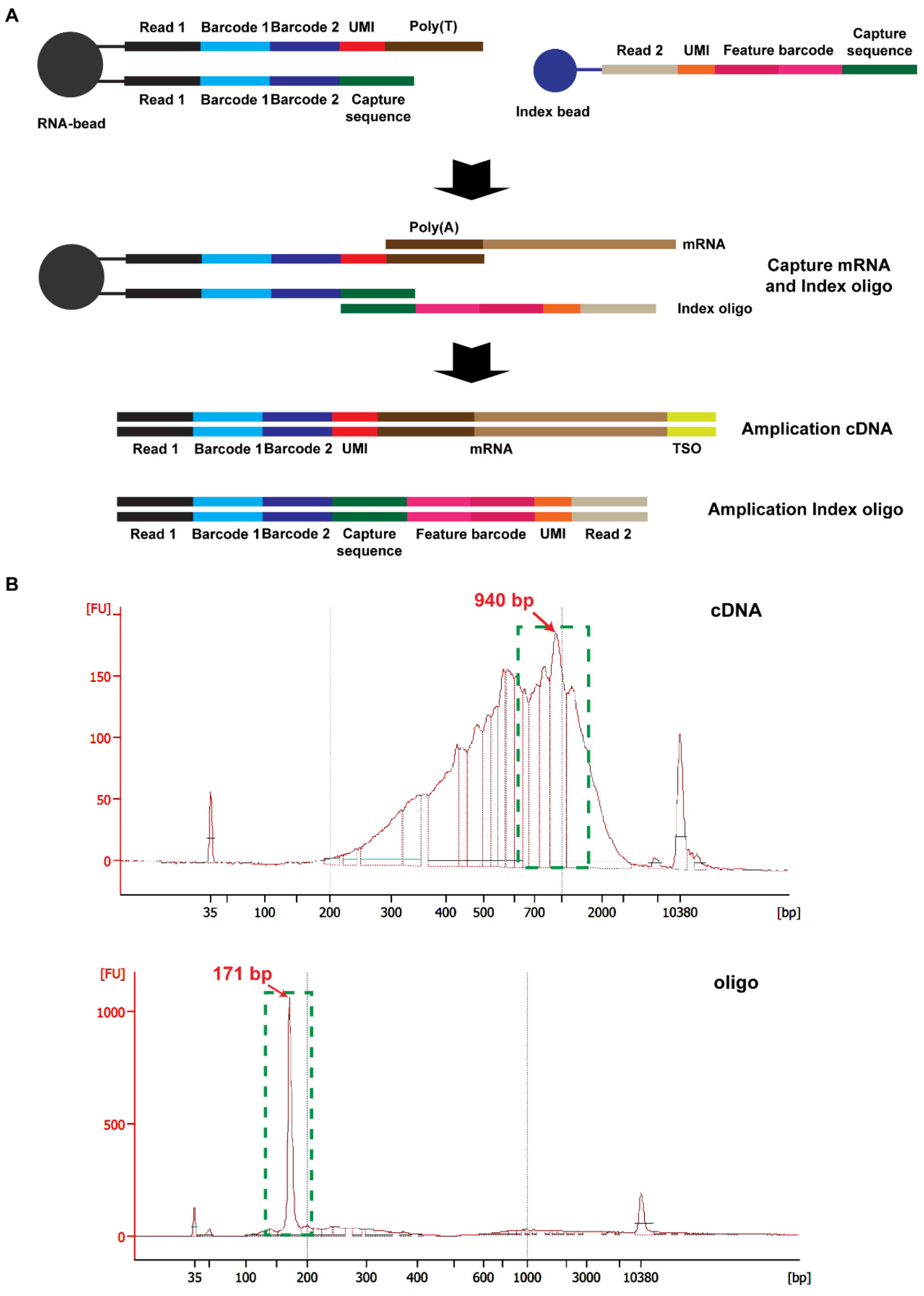

Figure S7. Principle and verification of nucleic acid capture. (A) Schematic diagram of library preparation includes capturing mRNA and index oligo, and amplifying cDNA and index oligo. There was a disulfide bond between the index bead and Read2, and the linker could be cleaved by tris(3-hydroxypropyl) phosphine or dithiothreitol. (B) Analysis of PCR production of cDNA and oligo. The main peak in the electrophoretogram of cDNA is ~1000 bp, and the electropherogram of oligo has an independent peak around 170bp.

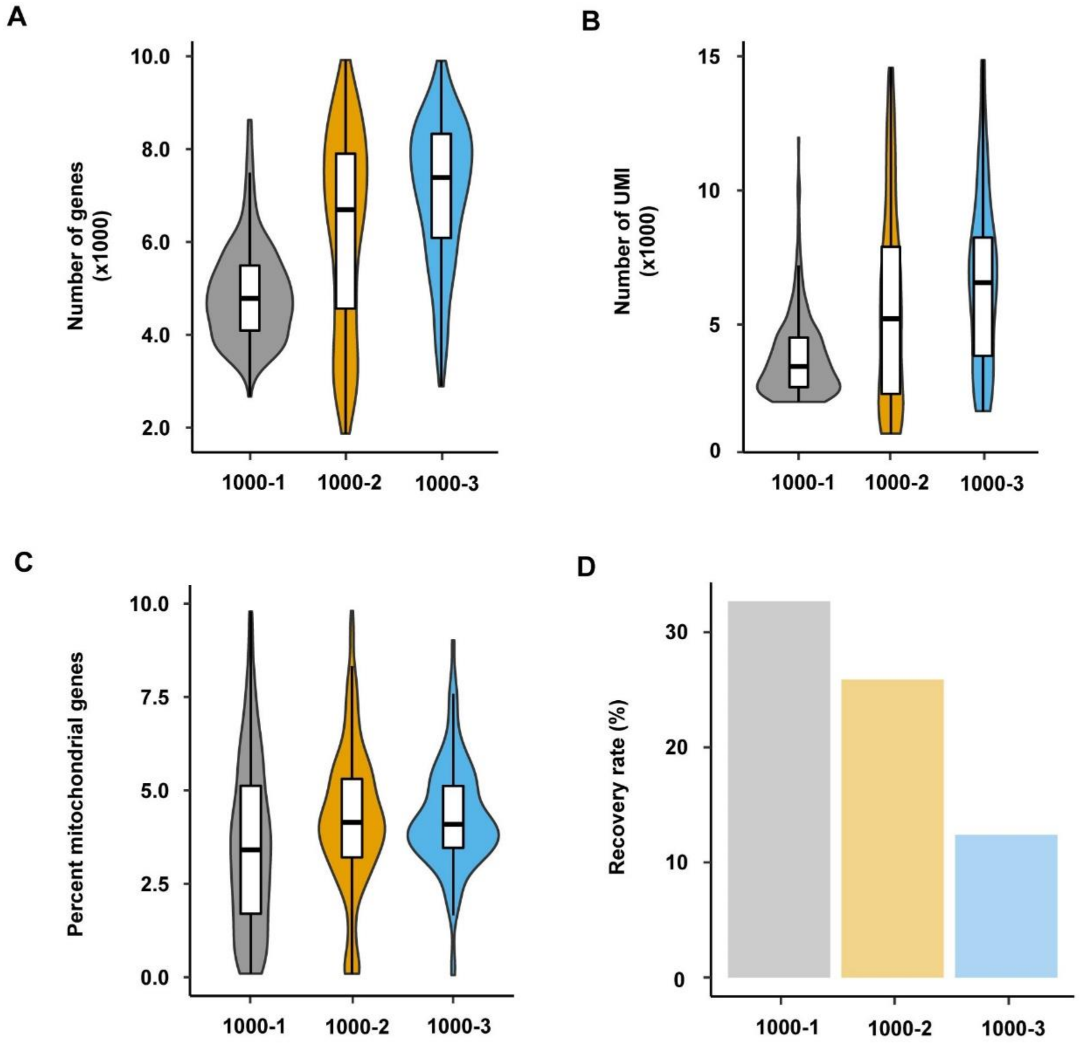

Figure S8. Qualification of 1000-cell samples by RNA sequencing. (A) The number of genes per cell for each sample. (B) Distribution of UMI number per cell for each sample. (C) The percentage of mitochondrial genes per cell. (D) The recovery rate of 1000-cell samples.

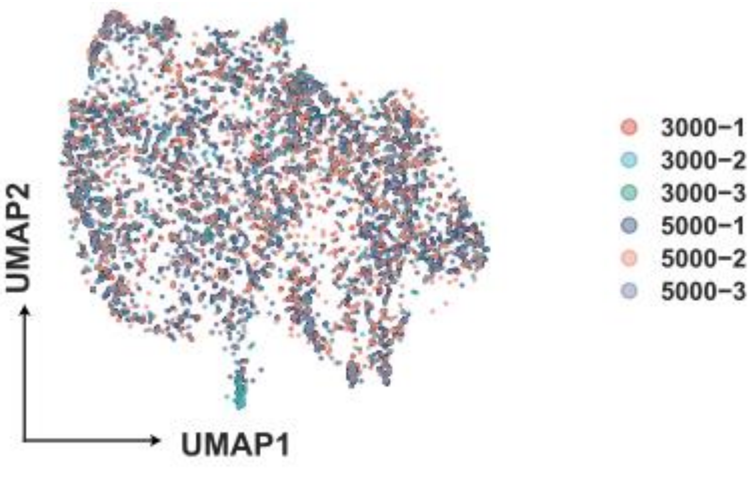

Figure S9. UMAP of cells from different samples. Cells of the six samples were presented on a UMAP plot after removing the batch effect.

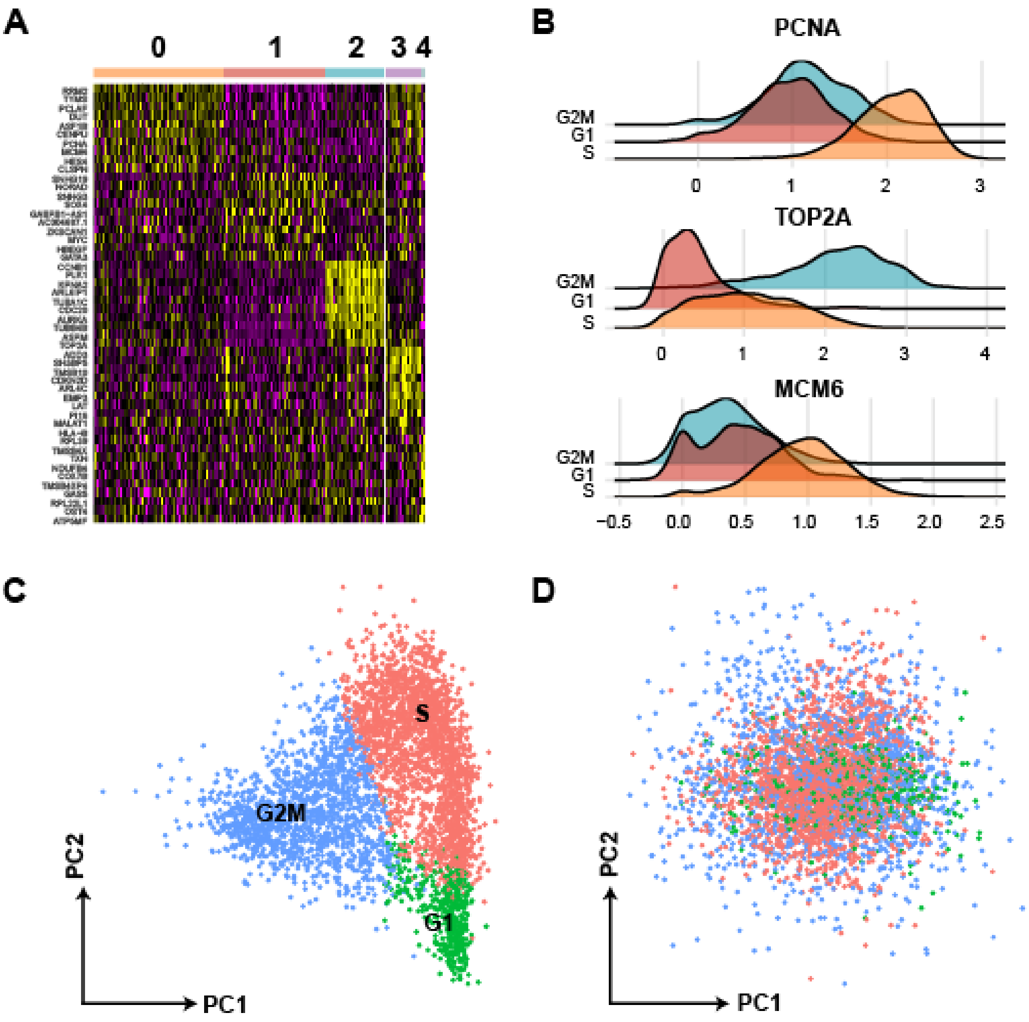

Figure S10. Cell cycle analyses of TCR-T cells. (A) Heatmap of top10 feature genes of each cluster. (B) Expression of PCNA, TOP2, and MCM6 of cells (C) PCA of TCR-T cells at different phases. (D) PCA of cells grouped by cell cycle markers with cell cycle genes regression.

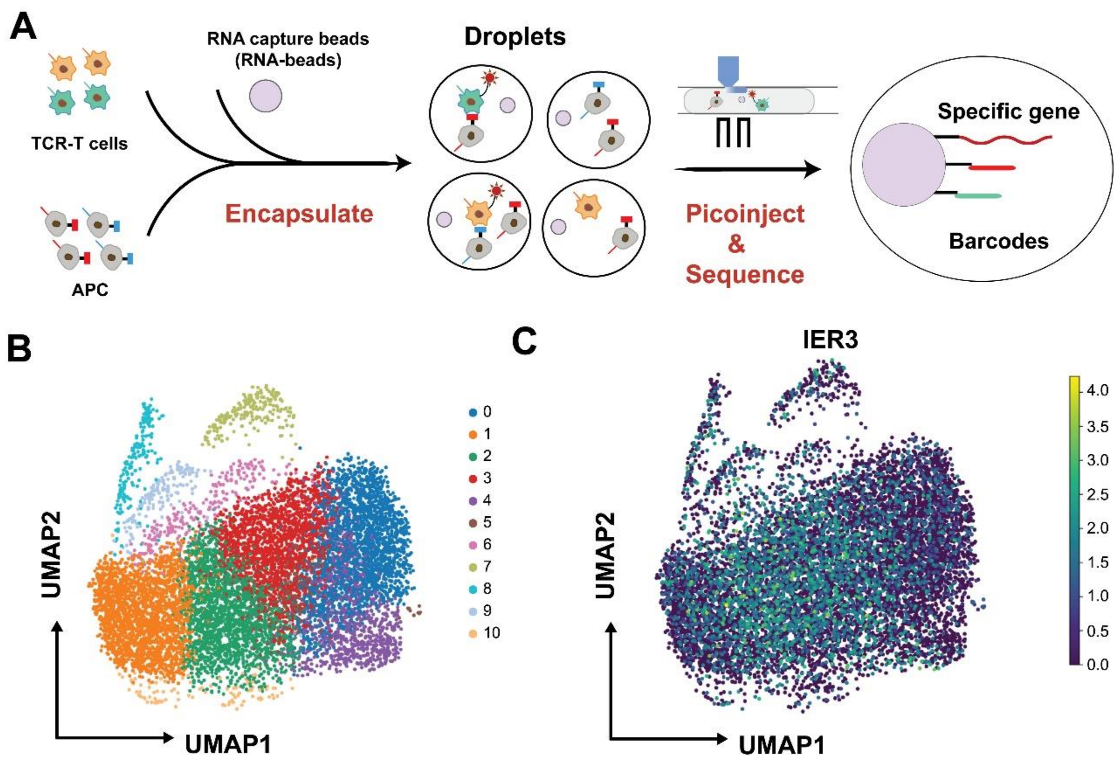

Figure S11. The strategy of the next work in antigen-specific TCR recognition. (A) A brief schematic diagram of experimental flow. In brief, TCR-T cells and APC cells were labeled with barcodes separately. Next, RNA-beads and cells were wrapped in droplets, and then the reagent injection introduced lysis buffer without destroying the pairing relationship of TCR-APC. After that, the recovery of molecular information, the library generation as well as the data analysis were used to confirm the corresponding barcodes of TCR-T and APC cells. Finally, the effect of TCR screening can be determined by a combination of barcodes and gene expression after the stimulation. (B) UMAP of all cells in droplets after picoinjection. (C) UMAP of IER3 involved in TCR activation.
